# *Trypanosoma cruzi* Infection Down-Modulates the Immunoproteasome Biosynthesis and the MHC Class I Cell Surface Expression in HeLa Cells

**DOI:** 10.1371/journal.pone.0095977

**Published:** 2014-04-21

**Authors:** Ricardo Camargo, Liliam O. Faria, Alexander Kloss, Cecília B. F. Favali, Ulrike Kuckelkorn, Peter-Michael Kloetzel, Cezar Martins de Sá, Beatriz D. Lima

**Affiliations:** 1 Departamento de Biologia Celular, Universidade de Brasília, Campus Universitário Darcy Ribeiro, Brasília, Distrito Federal, Brazil; 2 Institute für Biochimie, Charité-Universitätsmedizin Berlin, Berlin, Germany; Federal University of São Paulo, Brazil

## Abstract

Generally, *Trypanosoma cruzi* infection in human is persistent and tends to chronicity, suggesting that the parasite evade the immune surveillance by down regulating the intracellular antigen processing routes. Within the MHC class I pathway, the majority of antigenic peptides are generated by the proteasome. However, upon IFN-γ stimulation, the catalytic constitutive subunits of the proteasome are replaced by the subunits β1i/LMP2, β2i/MECL-1 and β5i/LMP7 to form the immunoproteasome. In this scenario, we analyzed whether the expression and activity of the constitutive and the immunoproteasome as well as the expression of other components of the MHC class I pathway are altered during the infection of HeLa cells with *T. cruzi*. By RT-PCR and two-dimensional gel electrophoresis analysis, we showed that the expression and composition of the constitutive proteasome is not affected by the parasite. In contrast, the biosynthesis of the β1i, β2i, β5i immunosubunits, PA28β, TAP1 and the MHC class I molecule as well as the proteasomal proteolytic activities were down-regulated in infected-IFN-γ-treated cell cultures. Taken together, our results provide evidence that the protozoan *T. cruzi* specifically modulates its infection through an unknown posttranscriptional mechanism that inhibits the expression of the MHC class I pathway components.

## Introduction

American trypanosomiasis or Chagas disease is caused by the haemoflagellate protozoan *Trypanosoma cruzi* and affects approximately 7–8 million people worldwide [1]. In humans, *T. cruzi* infection usually develops from an acute phase, characterized by high parasitemia and a robust immune response, to a clinically variable chronic phase. In this phase parasite proliferation is largely contained but the infection is persistent, particularly in the myocardium and smooth muscle, which may lead to the development of cardiac and digestive complications (for a review see [2]).

Although *T. cruzi* exhibits tropism to muscle cells in the mammalian host, the infective forms of the parasite, trypomastigotes, are highly motile and capable to infect different cell types [3]. After invasion, the parasites differentiate into amastigotes, escape from the parasitophorous vacuole to the cytoplasm and begin multiplication by binary fission [4]–[6]. Once *T. cruzi* has part of its life cycle in the host cell cytoplasm, it is known that in this context parasite antigens may be presented by immune and non-immune cells on surface-expressed major histocompatibility complex (MHC) class I molecules for recognition by CD8^+^ T lymphocytes [7], [8]. In murine models of infection, it has been shown that CD8^+^ T lymphocytes play crucial roles in the control of the *T. cruzi* parasitemia [9], [10]. The CD8^+^ T lymphocytes protect the host against *T. cruzi* through their cytolytic activity [11] and their production of interferon-γ (IFN-γ) and tumor necrosis factor-α (TNF-α), two pro-inflammatory cytokines known to be involved in infection control [12], [13]. Antigenic peptides presented to CD8^+^ T cells by MHC class I are generated mainly by the action of the proteasome, a multicatalytic complex responsible for the degradation of cytosolic and nuclear proteins generally poly-ubiquitylated. In the immune context, the proteasomes acting with downstream aminopeptidases generate peptide fragments of a size appropriate for transport by the transporter associated with antigen presentation (TAPs) into the endoplasmic reticulum for docking to the peptide binding groove of the MHC class I molecule [14]–[18].

Different types of proteasomes varying catalytic subunits and regulatory complexes are known in eukaryotes. The core 20S standard proteasome is a barrel-shaped particle composed of four heptameric stacked rings. The two outer rings consist of seven different but related α-subunits (α1-α7). They provide the proteasome structure, interact with regulatory factors and complexes, such as the 19S ATP-dependent regulator, and control the access of proteins into the catalytic chamber [19]. The two inner rings are each composed of seven different β subunits (β1-β7). The 20S standard proteasome has three major proteolytic activities defined as caspase-, trypsin- and chymotrypsin-like, with the corresponding catalytic sites being assigned to the subunits β1, β2 and β5, respectively [20]. In some cells of hematopoietic system, or during an immune response after IFN-γ or TNF-α stimulation, these three constitutively expressed subunits are replaced by the inducible subunits β1i/LMP2, β2i/MECL-1, and β5i/LMP7 to form the so-called immunoproteasome [21], [22]. The IFN-γ also induces the synthesis of the proteasome activator proteins PA28α and PA28β, components of the 11S regulatory complex [23]–[25]. The subunit replacements and the association of the 11S regulator to at least one end of the 20S core alter the cleavage pattern of the proteasome, optimizing the generation of small peptides for loading on the groove of MHC class I molecules [25]–[27]. These changes are also related to increase the production of immunogenic peptides compared to standard proteasome [28], [29].

Evidence of the significance of immunoproteasome in antigen processing came from studies showing that the overexpression of β1i, β2i, and β5i in cell lines enhanced the presentation of different viral epitopes, such as the NP118 epitope of the lymphocytic choriomeningitis virus nucleoprotein [30] or an epitope derived from the GagL protein of Moloney murine leukemia virus [31]. In addition, the deficiency in the immunosubunits β1i or β5i reduced the cytotoxic T lymphocyte repertoire and thus the efficiency of the immune response [32]–[34]. Furthermore, it was also shown that the deletion of β5i decreased the MHC class I cell surface expression by about 25–50% [32], [35].

Despite the demonstrated role of CD8^+^ T cells in defense against *T. cruzi* [10], [36], [37], the parasite persists for many years in the mammalian host, suggesting that *T. cruzi* escapes from the immune system by down-regulating the intracellular antigen processing routes. Given that MHC class I antigen presentation provides the basis for CD8^+^ T cells immunity, we decided to analyze if the expression and the proteolytic activity of the constitutive and the immunoproteasome as well as the expression of other components of the MHC class I pathway are altered during the infection of HeLa cells with the protozoan *T. cruzi*. Here, we report that the β1i, β2i, β5i immunosubunits as well as PA28β, TAP1 and the MHC class I molecule have their synthesis suppressed on the infected-IFN-γ-treated cell cultures. The proteasomal proteolytic activities were also affected by the infection. We demonstrate that this effect is not through general inhibition of the response to IFN-γ, but due to an unknown posttranscriptional regulation imposed by the parasite. Thus, our results bring new knowledge and insights about the mechanisms related to *T. cruzi* persistency, antigen presentation and immunopathogenesis. This could potentially have an impact on vaccines strategies and drugs development.

## Materials and Methods

### Cell lines and parasite cultures

HeLa, a human epithelial carcinoma non-immune cell line, was cultivated in standard DMEM (Dulbecco's modified Eagle's medium – Gibco, Life Technologies) supplemented with 10% FBS (Gibco, Life Technologies), 100 µg/mL ampicillin (Sigma-Aldrich) and 100 µg/mL streptomycin (Sigma-Aldrich) at 37°C in a humidified atmosphere containing 5% CO_2_.

The Y strain of *T. cruzi* [38] was used in all experiments. Trypomastigotes were grown and purified from cultures of a rat myoblast cell line (L6) as previously described [39].

### *In vitro* infection of cells with *T. cruzi* and IFN-γ treatment

Monolayers of HeLa (8×10^4^ cells/cm^2^) were obtained 24 h after plating 4×10^4^ cells/cm^2^ in DMEM-10% FBS on 80 cm^2^ flasks. Trypomastigotes were incubated with HeLa cells at 37°C for 24 h or longer at a parasite-host cell ratio of 40:1. At this ratio, 80–100% of the host cells presented amastigotes inside their cytoplasm after 24 h of parasite inoculation [40].

For induction of the proteasome immunosubunits as well as TAP1 and MHC class I molecule, cells were incubated with 90 U/mL of recombinant human IFN-γ (Calbiochem) for 24 h before or 24 h after the infection with *T. cruzi*.

After each treatment, cells were washed twice in PBS, detached with trypsin-versene solution (0.05% trypsin, 1× PBS, 2 mM EDTA), washed three times with cold PBS, and collected for proper processing and analysis.

### Experimental model

In order to study the effect of *T. cruzi* infection on the intracellular antigen processing routes in non-immune cells, HeLa cells were cultivated in five different conditions described as follows. First, cells were cultured without any treatment (designated as control). Second, cells were stimulated for 24 h with IFN-γ (named as IFN-γ). Third, cells were infected for 24 h with *T. cruzi* (Tc). Fourth, HeLa cells were previously infected with *T. cruzi* for 24 h and subsequently treated with IFN-γ for 24 h (Tc→IFN-γ). Fifth, cells were initially stimulated with IFN-γ for 24 h and then infected with *T. cruzi* for 24 h (IFN-γ→Tc). With this experimental model we simulated three distinct moments in an infection with *T. cruzi*: (a) invasion without immune response (Tc); (b) invasion with subsequent immune response (Tc→IFN-γ); (c) invasion in an environment with an established immune response (IFN-γ→Tc). This model was used throughout all the experiments, except in specified cases.

### RT-PCR and RT-qPCR Analyses

For semi-quantitative RT-PCR and quantitative real-time RT-qPCR analyses the RNA samples were isolated using TRIzol® reagent (Invitrogen) according to the manufacturer's instructions. The concentration and quality of the isolated RNA was determined using a NanoDrop Spectrophotometer (Thermo Scientific) and 1% agarose gel electrophoresis. For cDNA synthesis, 2 µg of total RNA were pre-treated with RQ1 RNase-Free DNase (Promega) and transcribed using the High Capacity cDNA Reverse Transcription Kit (Applied Biosystems) according to manufacturer's instructions.

For semi-quantitative RT-PCR, two microliters of cDNA were used for amplification in 30 µL PCR reaction containing 0.1 mM dNTPs (Invitrogen), 1.5 mM MgCl_2_, 1× PCR Buffer, 1.5 U Taq *Platinum* DNA polymerase (Invitrogen) and 0.3 (genes of proteasome)/0.1 (*GAPDH*) µM of each primer. The proteasomal and glyceraldehyde-3-phosphate dehydrogenase (*GAPDH*) primers were used for simultaneous amplification in one reaction tube. PCR conditions were as follows: 94°C/1 min; 94°C/30 s, 56°C/30 s, 72°C/40 s–30 cycles); 72°C/1 min. Ten microliters of the PCR products were analyzed in 1.2% agarose gels stained with ethidium bromide.

Real-time RT-qPCR reactions (final volume of 10 µL) contained 1× fast SYBR® Green Master Mix (Applied Biosystems), 0.25 µM forward-primer, 0.25 µM reverse-primer and 4 µL cDNA diluted 1:10 in UltraPure Water (Ambion). The amplification was performed on StepOne Plus Real-Time PCR System (Applied Biosystems), and SDS 2.3 software (Applied Biosystems) and Excel 2010 software (Microsoft) were used for data analysis and graphing, respectively. The expression of *GAPDH* and hypoxanthine phosphoribosyltransferase 1 (*HPRT1*) were used together as reference genes. The relative expression was calculated based on the 2^−ΔΔCT^ method [41], and the calibrator used was the IFN-γ treatment.

All primers used in semi-quantitative RT-PCR analysis are described in Ref. [40]. Primers used in real-time RT-qPCR for amplification of *GAPDH*, *HPRT1* and for the small subunit of the MHC class I molecule called β2-microglobulin (β2M) are described in Ref. [42]. Primers for the three human MHC class I alpha chain alleles, which are also called human leukocyte antigens (HLA) −A, −B and –C, are described in supporting information of Ref. [43]. The following primers were used in real-time RT-qPCR for amplification of immunoproteasome subunits and *TAP1*. mRNA accession numbers are indicated.

*α1*: fwd 5′-CATTTGAACAGACAGTGGAA-3′, rev 5′-TAGAGCAACAAGGTGAGC-3′; NM_002791.1.

*β1i*: fwd 5′-AGGAGGTCAGGTATATGGA, rev 5′- AATAGCGTCTGTGGTGAA-3′; NM_002800.4.

*β2i*: fwd 5′-CCCAAAATCTACTGCTGTG-3′, rev 5′- GTACCTGAAGAGCGTCTG-3′; NM_002801.2.

*β5i*: fwd 5′-ATATGTTCTCCACGGGTAG-3′, rev 5′- ATATTGACAACGCCTCCA-3′; NM_004159.4.

*PA28β*: fwd 5′-CTTTTCCAGGAGGCTGAGG-3′, rev 5′- CGGAGGGAAGTCAAGTCA-3′; NM_002818.2.

*TAP1*: fwd 5′-CTCATGTCCATTCTCACCATAGCCAG-3′, rev 5′-CAGCCCCAAACACCTCTCC-3′; NM_000593.

### Metabolic radiolabelling, immunoprecipitation, and two-dimensional gel electrophoresis

HeLa cells in standard culture conditions were exposed to *T. cruzi* (40 parasites per HeLa cell) and cultured for 24 h. In the twenty-first hour of culture, DMEM was replaced by methionine-deficient DMEM (Gibco, Life Technologies) and pulse chase was carried out with 100 μCi of [^35^S] methionine per mL (GE Healthcare Life Sciences). Cells were labeled for 3 h and then washed with PBS, harvested, and lysed on ice with 1% Triton X-100 in PBS in the presence of a protease inhibitor cocktail (Roche). The lysate was clarified by centrifugation at 15,000 g for 15 min at 4°C. The incorporation of radioactivity was determined by liquid scintillation counting (Beckman LS 7000) and the protein content was measured by Bio-Rad Protein Assay reagent (Bio-Rad) [44]. For proteasome immunoprecipitation, equal counts or 100 µg total lysates were incubated with mAb against human proteasome (p23K, p25K, p33K p27K, p29K and p31K) [45] and processed as described by Jackson et al. (1990) [46]. Immune complexes were precipitated using protein G-Sepharose beads (Fluka) and centrifugation. After extensive washing, immunoprecipitates were resuspended in a non-equilibrium pH gradient electrophoresis sample buffer and subjected to a two-dimensional gel electrophoresis using Ampholines pH 3.5–10 [47]. SDS-PAGE and radiography were carried out as previously described [48], [49]. The radiographs were scanned and the spots intensities determined in the Image Master Platinum software version 5.0 (Amersham, GE Healthcare Life Sciences). The control culture was subjected to the same described procedures. The identification of spots was performed by comparing the obtained images with proteomic human 20S proteasome data from Claverol et al. (2002) [50].

### Preparation of protein extracts

Protein extracts used in western blots and proteasome activity assays were both obtained by cell lysis in hypotonic buffer by sonication. Briefly, the cell pellet collected from the cell cultures was resuspended in 300 µL of cold hypotonic buffer (10 mM NaCl, 10 mM Tris-HCl pH 7.5, 5 mM MgCl_2_) supplemented with protease inhibitors (Roche) and incubated on ice for 5 min. Next, cells were lysed by sonication using an Ultrasonic Processor (Model GE 50T) in ice bath, 4 pulses of 20 s with intervals of 60 s in a range of 80%. The extracts were clarified by centrifugation at 12,000 g for 15 min at 4°C. The protein concentration was determined by Bio-Rad Protein Assay reagent (Bio-Rad) and then the extracts were stored at −80°C until use.

### Western Blotting

Equal amounts of total protein extracts (25-50 µg per lane) were separated on 13% SDS-PAGE gels and transferred to Immobilon-FL PVDF membranes as previously described [51]. Following protein transfer, membranes were blocked in 5% non-fat milk in TBS (25 mM Tris HCl pH 7.4, 137 mM NaCl) and incubated sequentially with primary and HRP-conjugated secondary antibodies. Results were visualized using ECL *plus* reagent (GE Healthcare Life Sciences) and radiography.

Rabbit polyclonal antibodies against β1i/LMP2, β5i/LMP7 and mouse monoclonal anti-MHC class I antibody (W6/32) were obtained from Abcam. Anti-MHC class I antibody recognizes the W6/32 antigenic determinant common to HLA-A, −B and −C of the human major histocompatibility complex. Mouse anti-PA28β was obtained from Cell Signaling and mouse anti-TAP1 from Rockland. Rabbit polyclonal antibodies anti-human β2i/MECL1 (K223) and α6/MCP20 were kindly provided by the Institute for Biochemistry, Humbolt University, Charité, Berlin. Mouse polyclonal antiserum anti-human α1 was obtained previously [45]. Mouse anti-*T. brucei* α-tubulin was obtained from Sigma-Aldrich and was used to recognize *T. cruzi* α-tubulin properly. Secondary antibodies were obtained from Biomol.

### Proteasome activity assay

The measurement of the proteasome proteolytic activity was performed using the Proteasome-Glo™ Chymotrypsin-Like, Trypsin-Like and Caspase-Like kits from Promega. The luminogenic substrates Z-LRR-aminoluciferin, Suc-LLVY-aminoluciferin e Z-nLPnLD-aminoluciferin, used to measure the trypsin-, chymotrypsin- and caspase-like activity, respectively, were prepared according to the manufacturer's protocol. Assays were performed at 25°C in opaque white 96-well plates and quantified by spectrophotometer (Spectra Max M5– Molecular Devices). To each well was added 1 µg of protein extract diluted in 10 mM HEPES (pH 7.6). The proportion of sample and reagents (v/v) was always 1:1 in a final volume of 50 µL. The specific activity of proteasome was estimated, for each treatment and substrate, through samples treated with the proteasome inhibitor MG-132 (50 µM per well) (Sigma Aldrich). Thus, the result of subtracting the intensity of luminescence of samples without MG-132 from those treated with the inhibitor refers only to proteasome activity.

### Flow cytometry and Immunofluorescence microscopy

HeLa cells were grown, treated and collected as described above. After counting, 1×10^6^ cells were blocked in 0.1% BSA for 15 min and incubated 30 min on ice with mouse anti-HLA class I antibody (W6/32) (Santa Cruz Biotechnologies) (1 µg Ab/10^6^ cells). Next, cells were washed in cold PBS and stained 30 min with PE-labelled anti-mouse antibody (Santa Cruz Biotechnologies) (0.5 µg Ab/10^6^ cells). Then, cells were washed and fixed in cold 1% paraformaldehyde in PBS. Flow cytometry was performed in FACSVerse (BD Biosciences) and the data analyzed in the BD FACSuite software (BD Biosciences). From each sample were acquired 2×10^4^ events and the MHC class I cell surface density was determined as median fluorescence intensity (MFI). For immunofluorescence microscopy, an aliquot from the same stained cells samples were mixed (1:1) with ProLong Gold antifade reagent with DAPI (Invitrogen), applied on a slide and covered with a coverslip. Prior to microscopy analysis, slides were incubated for 24 h in a dark room and then analyzed in the TCP SP5 confocal microscope (Leica Microsystems).

### Densitometry

The densitometric analysis were done using ImageJ software version 1.47 [52]. For quantification of the semi-quantitative RT-PCRs, the intensities of bands related to proteasome amplicons were normalized to the expression of the control gene (*GAPDH*). In the case of western blots, the experiments were normalized to the signal of the constitutive subunits α1 or α6.

### Statistics

All values presented in figures throughout the manuscript are means ± standard deviation of biological replicates. The number of replicates of each experiment is indicated in the figure legend. The significance of the results was determined by unpaired, two-tailed t-test and the indicated significance levels in the graphs are: ns – not significant, * p<0.05, ** p<0.01. All graphs and statistical analysis were performed in Excel 2010 software (Microsoft).

## Results

### The expression and composition of standard proteasome are not altered by *T. cruzi* infection

**Figure 1:**
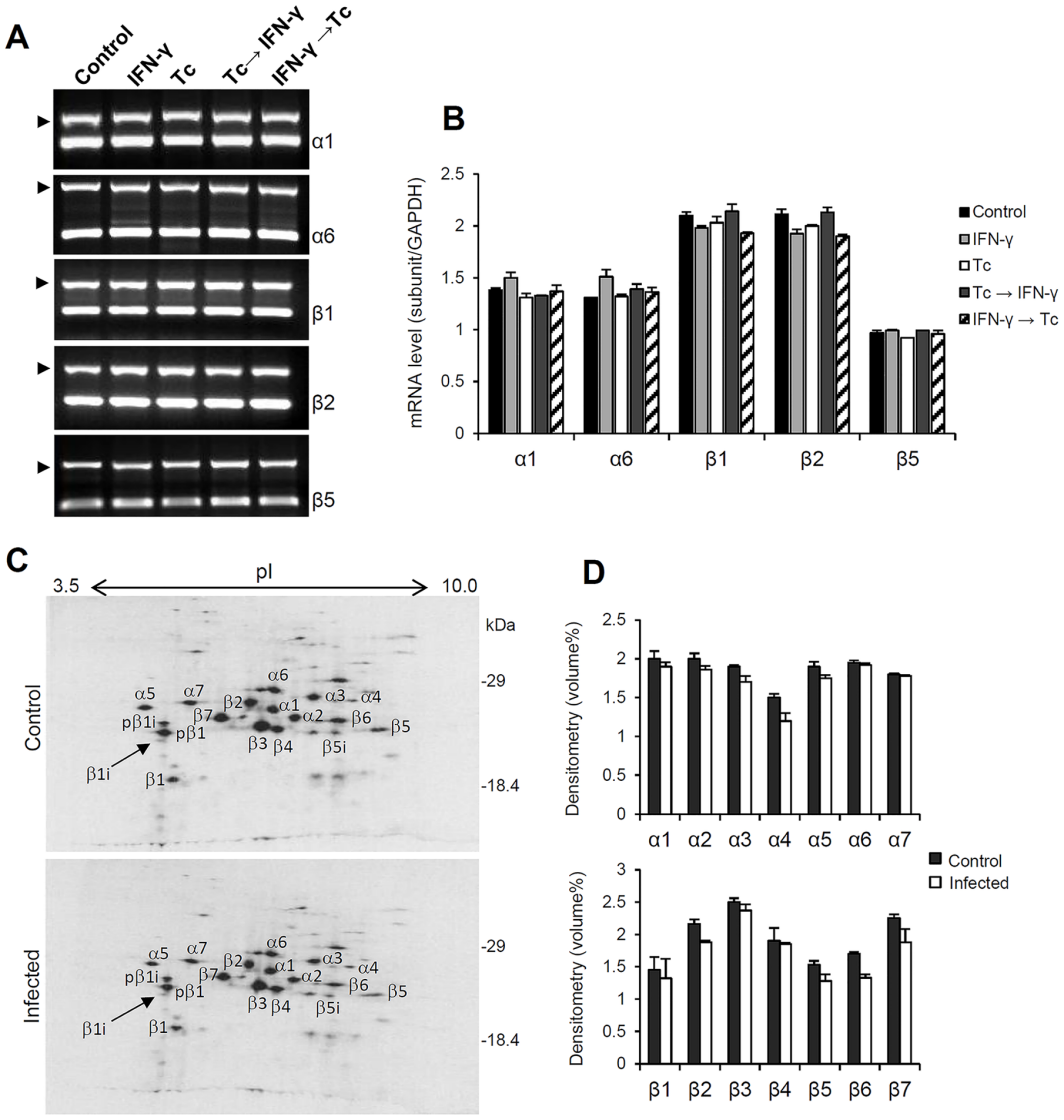
Analysis of mRNA levels and protein composition of HeLa constitutive proteasome during *T. cruzi* infection. Semi-quantitative RT-PCR analysis of α1, α6, β1, β2 and β5 expression were done using total RNA from HeLa cells treated with IFN-γ and/or infected with *T. cruzi*. (A) The PCR products were analyzed by electrophoresis in 1.2% agarose gels stained with ethidium bromide. The reactions were carried as duplex-PCR, using *GAPDH* as internal control (arrows). (B) mRNA levels were determined by densitometry and plotted using the expression of *GAPDH* as normalizer. Each value represents the mean ± standard deviation of three individual experiments. (C) Two-dimensional gels of immunoprecipitated proteasomes from HeLa uninfected and infected with *T. cruzi*. HeLa cells in standard culture conditions were exposed or not to *T. cruzi* and cultured for 24 h. In the twenty-first hour of culture, cells were metabolically labeled with [^35^S]-methionine for three hours. Cell lysates (100 µg) were immunoprecipitated with anti-human proteasome antibodies and analyzed by two-dimensional electrophoresis. Panel D show the protein levels of proteasome α and β subunits quantified by densitometry. Each value represents the mean ± mean deviation of two independent experiments.

Initially, we investigated whether *T. cruzi* affects the expression of HeLa constitutive proteasome. The mRNA levels of the α1, α6, β1, β2 and β5 subunits were determined by semi-quantitative RT-PCR analysis. As shown in figures 1A and 1B, the expression of these proteasomal subunits was not altered by the parasite. Thus, *T. cruzi* infection seems to have no effect on transcription of the constitutive proteasome genes. Furthermore, IFN-γ treatment also did not change these mRNA levels.

Next, at the protein level, we examined the impact of *T. cruzi* infection on constitutive proteasome composition. Immunoprecipitations of *in vitro* labeled proteasomes of infected or not infected HeLa cells were performed and analyzed by two-dimensional gel electrophoresis. The identification of spots was performed using as reference the two-dimensional electrophoretic map of human 20S proteasome described by Claverol et al. (2002) [50]. The abundance of the proteasome subunits and their composition pattern were practically identical between infected and uninfected cells (Fig. 1C–D). Taken together, the results of mRNA and protein expression indicate that the biogenesis of the constitutive proteasome is not affected by the parasite *T. cruzi*. These data are consistent with our previous results [40].

### Analysis of immunoproteasome mRNA levels during *T. cruzi* infection

**Figure 2:**
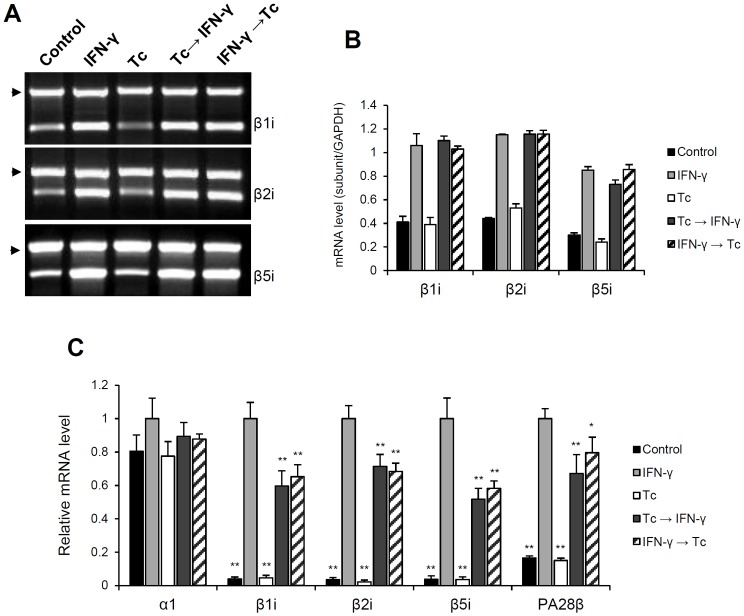
Analysis of immunoproteasome mRNA levels during *T. cruzi* infection. Semi-quantitative RT-PCR analysis of β1i, β2i and β5i expression were done using total RNA from HeLa cells treated with IFN-γ and/or infected with *T. cruzi*. (A) The PCR products were analyzed by electrophoresis in 1.2% agarose gels stained with ethidium bromide. The reactions were carried as duplex-PCR, using *GAPDH* as internal control (arrows). (B) mRNA levels were determined by densitometry and plotted using the expression of *GAPDH* as normalizer. Each value represents the mean ± standard deviation of three individual experiments. The abundance of α1, β1i, β2i, β5i and PA28β mRNAs were also determined by real time RT-qPCR. The relative expression of the transcripts was calculated by normalization with *GAPDH* and *HPRT1* housekeeping genes using the 2^−ΔΔCt^ method. (C) The mRNA levels were plotted relatively to “IFN-γ” experimental condition (HeLa treated 24 h with IFN-γ). Each value represents the mean ± standard deviation of three independent experiments.

In order to assess the effect of *T. cruzi* infection on immunoproteasome synthesis, we determined by semi-quantitative RT-PCR analysis the mRNA levels of the catalytic immunosubunits β1i, β2i and β5i (Fig. 2A–B). As expected, treatment of HeLa cells with IFN-γ induced the expression of the β-immunosubunits (lane IFN-γ). However, this induction was not affected by *T. cruzi* infection, regardless of the order of parasite inoculation and IFN-γ treatment (Tc→IFN-γ and IFN-γ→Tc). It is important to note that in HeLa cells the subunits β1i, β2i and β5i have a discrete basal expression, which is also not affected by infection (control and Tc).

To refine the mRNA quantification and determine accurately its relative abundance, we performed real-time RT-qPCR experiments (Fig. 2C). In addition to analyzing the expression of the α1, β1i, β2i and β5i subunits, we included the regulatory subunit PA28β. The calibrator used was the “IFN-γ” condition (HeLa treated 24 h with IFN-γ); therefore, all values shown in the graphs are relative to “IFN-γ” gene expression. Independent of infection, induction of transcription of the β1i, β2i, β5i subunits and also PA28β was observed after IFN-γ treatment. But, due to the higher resolution and greater sensitivity of the RT-qPCR technique, we noticed that after IFN-γ stimulation the mRNA levels of the infected cells were significantly lower than those of uninfected cells (Fig. 2C, Tc→IFN-γ and IFN-γ→Tc compared to IFN-γ). In these cell cultures, mRNA expression was decreased by up to 40% for β1i, 32% for β2i, 50% for β5i and 33% for PA28β. Even though the immunoproteasome expression was different from IFN-γ-treated uninfected cells the abundance of these transcripts was similar in both treatments (Tc→IFN-γ and IFN-γ→Tc). Relative quantification of the α1 subunit showed no difference between the five treatments. Its mRNA levels were not altered by IFN-γ treatment or by *T. cruzi* infection.

### *T. cruzi* infection prior to IFN-γ-treatment prevents the biosynthesis of HeLa immunoproteasome

**Figure 3:**
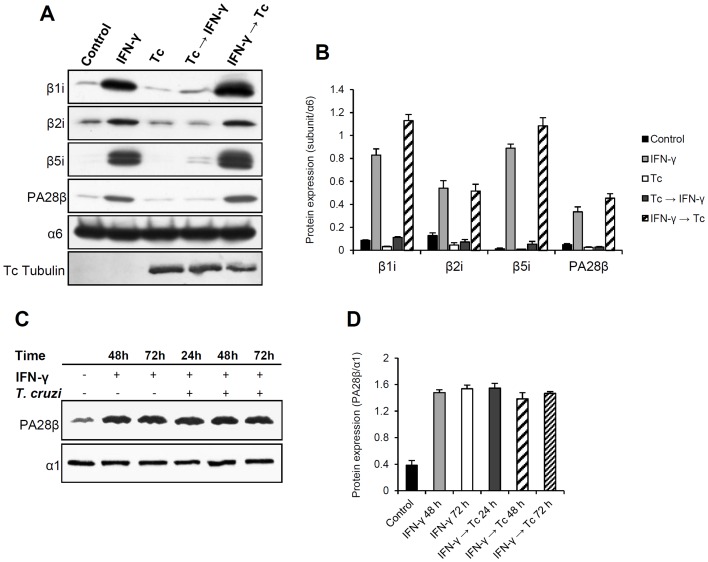
Analysis of immunoproteasome protein expression during *T. cruzi* infection. (A) Lysates (25–50 µg) of HeLa cells treated with IFN-γ and/or infected with *T. cruzi* were analyzed by western blot using anti-immunoproteasome subunits antibodies as indicated. (B) Protein levels were determined by densitometry and plotted using the expression of α6 subunit as experimental normalizer. Infection was confirmed using anti-tubulin antibody. (C) Western blot analysis of PA28β expression during different times of infection. HeLa cells were pre-treated with IFN-γ for 24 h and then infected with *T. cruzi* for 24, 48 and 72 h. (D) Protein levels were plotted using the expression of α1 as experimental normalizer. All values in this figure represent mean ± standard deviation of three individual experiments.

Since we have shown that the parasite has an impact on the transcription of IFN-γ-inducible subunits, it was of interest to evaluate the immunoproteasome protein levels during infection. For this purpose, western blot analysis was performed using antibodies against β1i, β2i, β5i and PA28β. Consistent with our previous results (Fig. 1C–D), the expression of the α6 subunit was not influenced by infection or by IFN-γ treatment (Fig. 3A). Under IFN-γ stimulation, the protein levels of uninfected HeLa cells (lane IFN-γ) doubled for β2i and PA28β and were ten times greater for β1i and β5i when compared to control. These levels remained the same or were a little higher when cells were treated with IFN-γ and subsequently infected with *T. cruzi* (IFN-γ→Tc). Interestingly, cells that were first infected with *T. cruzi* and then treated with IFN-γ (Tc→IFN-γ) exhibited the protein levels of the immunosubunits close to the control. We also observed that HeLa possesses a discrete basal expression of β1i, β2i, β5i and PA28β, however, these levels were slightly reduced with *T. cruzi* infection (Tc). It is important to note that once induced by IFN-γ, the protein levels of these subunits remained unchanged in the presence of the parasite (IFN-γ→Tc). Thus, these data suggest that *T. cruzi* does not degrade the immunosubunits and PA28β, but blocks their biosynthesis, given that the mRNAs levels of infected and IFN-γ treated cultures (Tc→IFN-γ) are similar to those of treated and infected cultures (IFN-γ→Tc) (Fig. 2). In other words, *T. cruzi* infection prior to IFN-γ-treatment prevents the induction of immunoproteasome subunits.

From these findings one important question arose: could time of infection and, consequently, parasite density influence the genesis of this inhibitory mechanism? This question emerged because in the experimental condition “Tc→IFN-γ”, in which immunoproteasome biosynthesis was inhibited, the infectious process lasted 48 h in total, whereas in “IFN-γ→Tc” it lasted 24 h. Knowing that cell invasion and amastigote replication, factors that increase parasite density, are directly related to time of infection, we performed experiments where HeLa cells were stimulated with IFN-γ for 24 h before being infected for 24 h, 48 h and 72 h. As control, cells were treated with IFN-γ for 24 h and left in culture for 48 h or 72 h. Cell lysates were analyzed by western blot using antibodies against PA28β and α1 subunit. These two proteins were chosen because the mechanism of inhibition acted upon PA28β expression, but not upon α1. Since α1 subunit expression was not affected by infection neither by IFN-γ-treatment, it was used as normalizer.

First, we observed that in the induced controls (IFN-γ: 48 h and 72 h) the PA28β protein level increased and did not change over 72 h after IFN-γ stimulation (Fig. 3C–D). According to protein turnover studies, the proteasome subunits, as well as the complex itself, have half-lives larger than two days, reaching up to 15 days depending on the cell type and tissue analyzed [53]–[56]. The expression of PA28β observed in the control was similar to the cultures infected with *T. cruzi*. They did not show any decay up to 72 h. Thus, we conclude that longer time of infection (with consequent increase in cytoplasmic parasite density) is not related to triggering the inhibitory phenomenon observed in figure 3A and is probably due to the presence of the parasite before the IFN-γ stimulation.

*T. cruzi* infection alters the proteolytic activity of the host proteasome.

**Figure 4:**
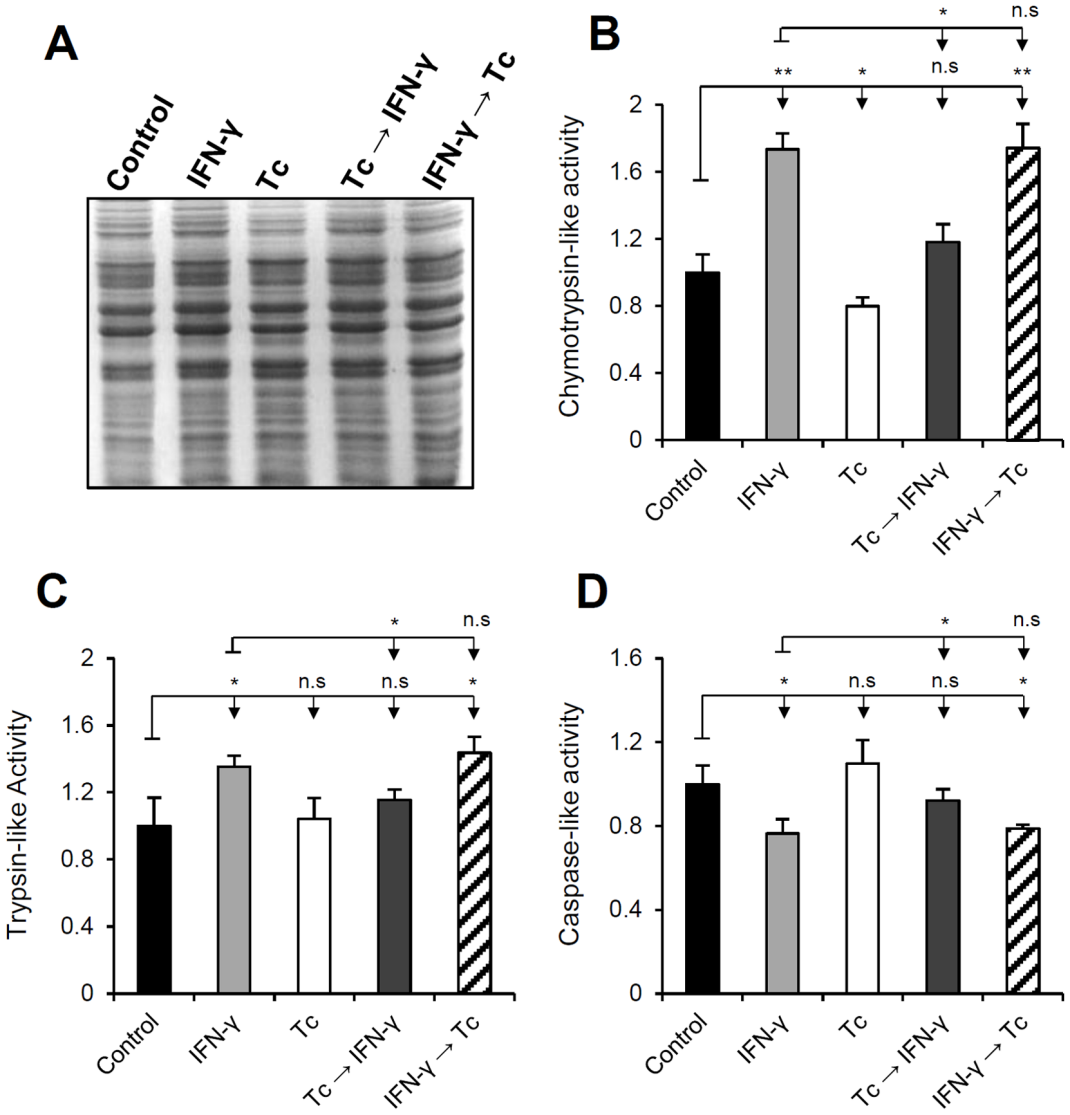
Effect of *T. cruzi* infection on proteasome proteolytic activities. Proteolytic assays were performed using extracts of HeLa cells treated with IFN-γ and/or *T. cruzi*-infected combined with luminogenic substrates specific for chymotrypsin-, trypsin- and caspase-like proteasome activities. The specific activity of the proteasome was estimated, for each treatment and substrate, through samples treated with the proteasome inhibitor MG-132. Mean of luminescence intensities were plotted relatively to the control. Prior to the catalytic assays, cell lysates (20 µg/lane) were analyzed by SDS-PAGE 13% stained with coomassie blue (A). Gels were used as loading control. (B) Chymotrypsin- (C) trypsin- and (D) caspase-like assays. Each value represents the mean ± standard deviation of three individual experiments and each sample was analyzed in triplicates.

Knowing that the 20S proteasome has three major proteolytic activities, defined as chymotrypsin-, trypsin- and caspase-like, we evaluated its catalytic profile during infection in order to relate the expression of the immunosubunits with the magnitude of their activities, especially in the intriguing case of the “Tc→IFN-γ” experimental condition. To this end, enzymatic assays were performed using cell extracts (Fig. 4A), obtained according to the proposed experimental model, combined with luminogenic substrates specific for each proteasomal activity.

Regarding the chymotrypsin-like activity (Fig. 4B), we observed that after IFN-γ stimulation the hydrolysis of the luminogenic substrate was increased approximately 70% in uninfected cells (IFN-γ). The same value was found in the “IFN-γ→Tc” experimental condition. In the “Tc→IFN-γ” condition the catalytic level was close to the control (18% higher), almost returning to basal activity of constitutive and immunoproteasome. In cell cultures only infected with *T. cruzi* (Tc), the peptide hydrolysis decreased about 20% when compared to the control. In the trypsin-like assays (Fig. 4C) the differences between the experimental conditions were not so pronounced as in the chymotrypsin tests. IFN-γ treatment increased 35% the substrate hydrolysis in uninfected cells (IFN-γ) and 40% in IFN-γ-treated-infected cultures (IFN-γ→Tc). In the other two treatments (Tc and Tc→IFN-γ), values were similar to the control. These results are in agreement with the protein levels shown in figure 3A, suggesting a direct relation between proteolysis and expression of the immunoproteasome subunits responsible for the chymotryptic and tryptic activities. In this particle, chymotrypsin-like activity is assigned to β1i and β5i subunits, and the trypsin-like to β2i. As noticed, once immunoproteasome is induced these two catalytic activities increased and were not affected by *T. cruzi* infection (IFN-γ and IFN-γ→Tc), different from the “Tc→IFN-γ” condition.

In caspase-like assays (Fig. 4D), in the treatments where the immunoproteasome synthesis occurred the substrate hydrolysis were reduced about 20% (IFN-γ and IFN-γ→Tc). It was expected because after IFN-γ stimulation the β1 subunit is replaced by β1i during proteasome neosynthesis. This change increases the proteasomes' capacity to cleave small peptides after hydrophobic residues instead of cleavage after acidic residues [57]. As observed in figure 1, the expression of β1 and its incorporation into mature particles were not altered during *T. cruzi* infection. So, the caspase-like activity was also not affected by the parasite since the peptide hydrolysis levels were similar to the control (lane Tc). In the treatments where the infection suppressed the immunoproteasome synthesis (Tc→IFN-γ) the peptide hydrolysis levels were also similar to the control. Thus, we conclude that *T. cruzi* infection does not affect the caspase-like activity, but the induction of the immunoproteasome does.

### The expression of TAP1 and MHC class I molecule was also affected by *T. cruzi* infection

**Figure 5:**
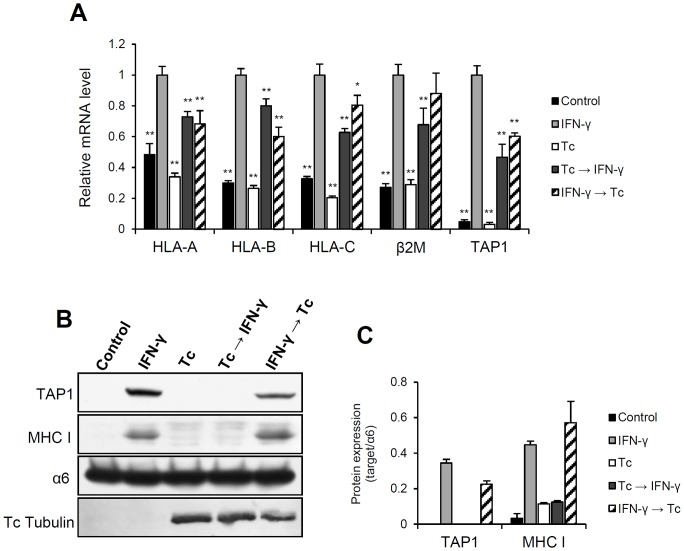
Quantification of mRNA and protein expression of TAP1 and MHC class I molecule during *T. cruzi* infection. (A) The abundance of *TAP1*, *β2M* and *HLA* mRNAs were determined by real-time RT-qPCR using the total RNA from HeLa cells treated with IFN-γ and/or infected with *T. cruzi*. The relative expression of the transcripts was calculated by normalization with *GAPDH* and *HPRT1* housekeeping genes using the 2^−ΔΔCt^ method. The mRNA levels were plotted relatively to “IFN-γ” experimental condition (HeLa treated 24 h with IFN-γ). Each value represents the mean ± standard deviation of three independent experiments. (B) Lysates (25–50 µg) of HeLa cells treated with IFN-γ and/or infected with *T. cruzi* were analyzed by western blot using human anti-TAP1 and anti-MHC class I antibodies as indicated. Infection was confirmed using anti-tubulin antibody. (C) Protein levels were determined by densitometry and plotted using the expression of α6 subunit as experimental normalizer. Each value represents the mean ± standard deviation of three independent experiments.

Once it was demonstrated that infection with *T. cruzi* prevents the host immunoproteasome biosynthesis, it was of interest to assess the expression of other important components of MHC class I pathway. Thus, we analyzed the MHC class I molecule itself and TAP1 expression. The transcripts were quantified by real-time RT-qPCR and the protein expression determined by western blot (Fig. 5).

Similar to the results obtained for the immunoproteasome, transcription of TAP1, β2M and MHC class I was induced after IFN-γ stimulation independent of the parasite inoculation (Fig. 5A). However, the mRNA levels of infected cells were significantly lower than that of uninfected cells (Tc→IFN-γ and IFN-γ→Tc). Nevertheless, there was induction and the abundance of these transcripts was almost the same in both treatments. Although reduced, these levels were sufficient for a detectable protein expression in the “IFN-γ→Tc” experimental condition (Fig. 5B–C). In contrast, the expression of MHC class I molecule and TAP1 in the infected-IFN-γ-treated cultures (Tc→IFN-γ) were close to basal levels. So, in our experimental model, TAP1 and MHC class I molecule exhibited the same behavior as the immunoproteasome subunits.

**Figure 6:**
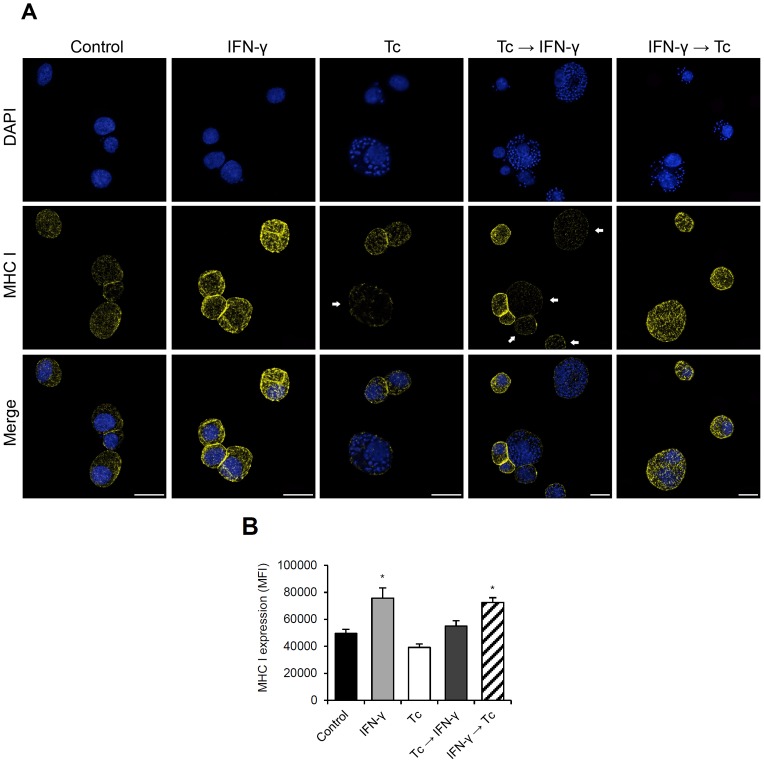
Effect of *T. cruzi* infection on MHC class I cell surface expression. (A) Immunofluorescence microscopy of HeLa cells treated with IFN-γ and/or *T. cruzi*-infected stained with human anti-MHC class I antibody (yellow) and DAPI (blue). The arrows highlight the infected cells that clearly had the MHC class I expression down-modulated by *T. cruzi* infection. Bars  = 25 µm. To precisely quantify the MHC class I cell surface expression, samples used in microscopy were analyzed by flow cytometry. (B) The protein expression was determined as median fluorescence intensity (MFI). Each value represents the mean ± standard deviation of three independent experiments.

To corroborate these results and evaluate the MHC class I cell surface expression, we performed immunofluorescence microscopy and flow cytometry analysis of the five proposed experimental conditions (Fig. 6). To precisely quantify the fluorescence intensity, samples used in microscopy were the same as used in flow cytometry. In addition to anti-MHC class I antibody, cells were stained with DAPI in order to detect the parasites and rate the infection density through microscopy. Consistent with the protein levels observed in the western blot analysis (Fig. 5), cell surface expression of MHC class I molecule was significantly lower (20%) in infected cells (Tc) compared to control (Fig. 6A–B). It was also reduced (40% lower than “IFN-γ” condition) where the infection occurred before the IFN-γ treatment (Fig. 6A–B, Tc→IFN-γ). In the images of this experimental condition (Fig. 6A, Tc→IFN-γ) the difference in MHC class I cell surface expression can be easily visualized when comparing the infected cells (highlighted by arrows) to uninfected or poorly infected HeLa cells. But, in cell cultures where the infection occurred after the IFN-γ stimulation (IFN-γ→Tc) the MHC class I surface expression was not affected after 24 h of parasite inoculation. In this condition, as well as in uninfected cells, IFN-γ treatment increased by 48% the amount of MHC class I molecule (Fig. 6B, IFN-γ and IFN-γ→Tc).

## Discussion

Here, we studied the impact of *T. cruzi* infection on the intracellular MHC class I antigen processing routes in HeLa cells. Our results show that the biosynthesis of the immunoproteasome subunits β1i, β2i, β5i as well as PA28β, TAP1 and MHC class I molecule were down-regulated by the parasite.

Since the generation of cytoplasm-derived antigenic peptides is assigned to both immuno- and standard proteasome, we initially investigated whether *T. cruzi* affects the expression and composition of HeLa constitutive proteasome. We found that infection with *T. cruzi* had no effect on the transcription and protein expression of the constitutive proteasome subunits. In contrast, upon IFN-γ stimulation the mRNA levels of the IFN-γ-inducible immunoproteasome subunits were decreased in infected cells in comparison to uninfected cells. This reduction may be related to the depletion of the IFN-γ membrane receptors, possibly caused by endocytic processes during parasite invasion, decreasing the cytokine stimulatory effect. Moreover, it may also be related to the density of amastigotes in host the cell cytoplasm, which may disturb cell signaling pathways or cellular metabolism.

At the protein level, we found that *T. cruzi* infection prior to IFN-γ-treatment prevents the biosynthesis of immunoproteasome subunits β1i, β2i, β5i, also PA28β, TAP1 and MHC class I molecule. Interestingly, the mRNAs levels of the infected and IFN-γ-treated cultures (Tc→IFN-γ) were similar to those of the IFN-γ-treated and infected cultures (IFN-γ→Tc), but the protein levels were quite different. After IFN-γ stimulation, the infection did not alter the proteasome immunosubunits, PA28β, TAP1 and MHC class I protein levels, indicating that *T. cruzi* does not degrade these proteins. On the other hand, in HeLa cultures where infection occurred before the cytokine treatment the expression of these proteins was close to the basal levels. Since the mRNA levels of these two experimental conditions (Tc→IFN-γ and IFN-γ→Tc) were the same but the protein levels differed, we propose that *T. cruzi* infection blocks the expression of the immunoproteasome subunits, PA28β, TAP1 and MHC class I molecule by an unknown posttranscriptional control. We suggest that this is due to specific inhibition of protein synthesis possibly caused by mRNA cytolocalization or impairment on translation initiation. Analyzing the PA28β expression, we demonstrated that triggering of this inhibitory phenomenon was independent of time of infection and consequently of parasite density, but due to *T. cruzi* inoculation before IFN-γ treatment preventing the protein translation. To our knowledge, this represents the first evidence that an intracellular protozoan parasite modulates its infection through a posttranscriptional mechanism that affects the translation of host proteins favoring its escape from the immune response.

Beside the immunoproteasome expression, we also analyzed its proteolytic activities during *T. cruzi* infection. In the enzymatic assays, in accordance with the protein levels, we found a direct relation between proteolysis and the expression of the immunoproteasome subunits responsible for the chymotryptic and tryptic activities. Since β1i and β5i had their protein synthesis down-regulated in infected-IFN-γ-treated cultures (Tc→IFN-γ), the chymotrypsin-like activity was decreased close to basal levels. The same occurred to the β2i, thus, the trypsin-like activity was also down-regulated by the parasite. The trypsin-like activity and mainly chymotrypsin-like activity seem to be the most relevant to the generation of antigenic peptides. This is because it is thought that cleavage after hydrophobic and basic amino acid residues produces peptides optimal for fitting into the cleft of the MHC class I molecule [25]–[27]. Also, the association of the PA28 regulator to one end of the 20S immunoproteasome core is related to increase the production of immunogenic peptides [28], [29]. Therefore, the down-modulation of PA28β and the β-immunosubunits synthesis during *T. cruzi* infection may be related to the poor quantity and quality of the generated immunogenic peptides, factors that could limit the recognition of the host cell by CD8^+^ T lymphocytes preventing an effective immune response.

Two works strengthen the hypothesis about the implications of ubiquitin-proteasome system in antigen presentation and parasite persistency during the *T. cruzi* infection. Chou et al. (2008) [58] testing a DNA vaccine against an epitope of *T. cruzi* reinforced the idea that the immunoproteasome and their regulator PA28 are essential for the protection of mice against the parasite. Bergeron et al. (2008) [59] showed that the infection of murine macrophages reduces the synthesis of immunoproteasome and MHC class I expression via SAPK/JNK signaling pathway, possibly through a transcriptional mechanism. Other study reported that MHC class I cell surface expression is down-regulated by *T. cruzi* [60], however, during infection of immune cells.

Since the proteasome is the key protease generating peptides for the MHC class I antigen presentation, not only *T. cruzi*, but many other infectious microorganisms and specially viruses have developed strategies to evade the ubiquitin-proteasome system hindering the recognition of these pathogens by the immune cells. For example, protein X of hepatitis B virus interacts with the α7 subunit of the proteasome and inhibits the trypsin- and chymotrypsin-like activities [61]. Other works showed that cells infected with adenovirus type 12E1A and HPV type 18 E7 exhibited a decrease in both mRNA and protein levels of TAP1 and TAP2, β1i and β5i [62]–[64]. Regarding the MHC class I molecule, its down-regulation has been observed as consequence of virus infection [65]–[68], as well as its up-regulation [69]–[71].

Despite the evidence for an apparently vigorous *T. cruzi*-specific CD8^+^ T-cell response [10], [36], [37], the vast majority of hosts fail to completely clear the parasite loading, reflecting the chronicity of the disease. So, our results bring new knowledge about the antigen presentation process during the *T. cruzi* infection and could be one clue for the mechanisms of parasite persistency and an explanation for the suboptimal CD8^+^ T lymphocyte response. Maybe, this is because the recognition of the infected cells by the CD8^+^ T lymphocyte is compromised by the lack of immunogenic epitopes derived from immunoproteasome activity. However, other studies should be undertaken to precisely elucidate the inhibitory mechanism induced by the parasite. It is also of interest to verify the immunoproteasome regulation in other cell types for which the parasite shows a high degree of tropism such as cells found in heart, nervous or digestive tissues and also perform *in vivo* studies.
